# Dynamic Encoding of Acoustic Features in Neural Responses to Continuous Speech

**DOI:** 10.1523/JNEUROSCI.2383-16.2017

**Published:** 2017-02-22

**Authors:** Bahar Khalighinejad, Guilherme Cruzatto da Silva, Nima Mesgarani

**Affiliations:** Department of Electrical Engineering, Columbia University, New York, New York 10027

**Keywords:** EEG, event-related potential, phonemes, speech

## Abstract

Humans are unique in their ability to communicate using spoken language. However, it remains unclear how the speech signal is transformed and represented in the brain at different stages of the auditory pathway. In this study, we characterized electroencephalography responses to continuous speech by obtaining the time-locked responses to phoneme instances (phoneme-related potential). We showed that responses to different phoneme categories are organized by phonetic features. We found that each instance of a phoneme in continuous speech produces multiple distinguishable neural responses occurring as early as 50 ms and as late as 400 ms after the phoneme onset. Comparing the patterns of phoneme similarity in the neural responses and the acoustic signals confirms a repetitive appearance of acoustic distinctions of phonemes in the neural data. Analysis of the phonetic and speaker information in neural activations revealed that different time intervals jointly encode the acoustic similarity of both phonetic and speaker categories. These findings provide evidence for a dynamic neural transformation of low-level speech features as they propagate along the auditory pathway, and form an empirical framework to study the representational changes in learning, attention, and speech disorders.

**SIGNIFICANCE STATEMENT** We characterized the properties of evoked neural responses to phoneme instances in continuous speech. We show that each instance of a phoneme in continuous speech produces several observable neural responses at different times occurring as early as 50 ms and as late as 400 ms after the phoneme onset. Each temporal event explicitly encodes the acoustic similarity of phonemes, and linguistic and nonlinguistic information are best represented at different time intervals. Finally, we show a joint encoding of phonetic and speaker information, where the neural representation of speakers is dependent on phoneme category. These findings provide compelling new evidence for dynamic processing of speech sounds in the auditory pathway.

## Introduction

When listening to speech, we have the ability to simultaneously extract information about both the content of the speech and the identity of the speaker. We automatically accomplish these parallel processes by decoding a multitude of cues encoded in the acoustic signal, including distinctive features of phonemic categories that carry meaning as well as identifiable features of the speaker, such as pitch, prosody, and accent (Stevens, 2000; Ladefoged and Johnson, 2010). Despite the extensive research to model and describe these processes, we still have no comprehensive and accurate framework for the transformation and representation of speech in the human brain (Poeppel, 2014). Recent invasive human neurophysiology studies have demonstrated the encoding of phonetic features in higher-level auditory cortices (Chan et al., 2014; Mesgarani et al., 2014). However, invasive recordings are limited to confined brain regions and are therefore impractical for studying the neural representation of acoustic features over time as speech sounds propagate through the auditory cortex (Hickok and Poeppel, 2007; Formisano et al., 2008).

Electroencephalography (EEG) has been used extensively in speech and language studies because it can measure the activity of the whole brain with high temporal resolution (Kaan, 2007). EEG studies of speech perception are primarily based on event-related potentials (ERPs; Osterhout et al., 1997). For example, ERPs have been used to study the encoding of acoustic features in response to isolated consonant–vowel pairs, showing a discriminant encoding at multiple time points (e.g., P1–N1–P2 complex) and locations (i.e., frontocentral and temporal electrodes; Picton et al., 1977; Phillips et al., 2000; Näätänen, 2001; Ceponiene et al., 2002; Tremblay et al., 2003; Martin et al., 2008). In addition, ERPs have been used in studies of higher-level speech units, such as word segmentation (Sanders and Neville, 2003) and multiscale hierarchical speech processing (Friederici et al., 1993; Kaan et al., 2000; Patel, 2003).

Nonetheless, ERP approaches suffer from unnatural experimental constraints (for example, requiring isolated, nonoverlapping events; Luck, 2014), which may result in only partial engagement of the underlying cortical circuits (Overath et al., 2015). As a result, these findings are not definitive enough to be useful in making generalizations applicable to more naturalistic settings. Several recent studies have examined EEG responses to continuous speech by correlating the responses with the speech envelope (Luo and Poeppel, 2007; Aiken and Picton, 2008; Kerlin et al., 2010; Kong et al., 2015) and by regressing the neural responses against the speech envelope (Lalor et al., 2009) or against the phonetic features and phonemes (Di Liberto et al., 2015). To study the precise temporal properties of neural responses to acoustic features, we propose an ERP method, where the events are the instances of phonemes in continuous speech. Specifically, we calculated the time-locked responses to phoneme instances and examined the representational properties of phonetic and speaker information in EEG signals. Moreover, we compared the similarity patterns of phonemes in acoustic and neural space over time. Finally, we examined the joint encoding of phonetic and speaker information and probed the phoneme-dependent representation of speaker features.

## Materials and Methods

### 

#### 

##### Participants.

Participants were 22 native speakers of American English with self-reported normal hearing. Twenty were right-handed. Twelve were males. Ten were females.

##### Stimuli and procedure.

EEG data were collected in a sound-proof, electrically shielded booth. Participants listened to short stories with alternating sentences spoken by a male and a female speaker; we alternated sentences to normalize time-varying effects such as direct current (DC) drift on speaker-dependent EEG responses. The stimuli were presented monophonically at a comfortable and constant volume from a loudspeaker in front of the subject. Five experimental blocks (12 min each) were presented to the subject with short breaks between each block. Subjects were asked to attend to the speech material. To assess attention, subjects were asked three questions about the content of the story after each block. All subjects were attentive and could correctly answer >60% of the questions. Participants were asked to refrain from movement and to maintain visual fixation on the center of a crosshair placed in front of them. All subjects provided written informed consent. The Institutional Review Board of Columbia University at Morningside Campus approved all procedures.

##### Recording.

EEG recordings were performed using a g.HIamp biosignal amplifier (Guger Technologies) with 62 active electrodes mounted on an elastic cap (10–20 enhanced montage). EEG data were recorded at a sampling rate of 2 kHz. A separate frontal electrode (AFz) was used as ground and the average of two earlobe electrodes were used as reference. The choice of earlobe as reference in studies of auditory-evoked potentials (AEPs) is motivated by the highly correlated activity across electrodes, which makes common reference averaging unsuitable (Rahne et al., 2007). EEG data were filtered online using a 0.01 Hz fourth-order high-pass Butterworth filter to remove DC drift. Channel impedances were kept below 20 kΩ throughout the recording.

##### Estimation of the acoustic spectrogram.

The time–frequency auditory representation of the speech stimuli was calculated using a model of the peripheral auditory system (Chi et al., 2005). The model consists of three stages: (1) a cochlear filter bank consisting of 128 asymmetric filters equally spaced on a logarithmic axis, (2) a hair cell stage consisting of a low-pass filter and a nonlinear compression function, and (3) a lateral inhibitory network consisting of a first-order derivative along the spectral axis. Finally, the envelope of each frequency band was calculated to obtain a two-dimensional time–frequency representation that simulates the pattern of activity on the auditory nerve (Wang and Shamma, 1994).

##### Preprocessing.

EEG data were filtered using a zero-lag, finite-impulse response bandpass filter with cutoff frequencies of 2 and 15 Hz (Delorme and Makeig, 2004). The frequency range was determined by measuring the average power of the phoneme-related potential (PRP) at different frequencies. This measurement showed that the PRP peaks at 8 Hz (the syllabic rate of speech). For each subject, we normalized the neural response of each EEG channel to ensure zero mean and unit variance.

##### PRP.

**Figure 1.**
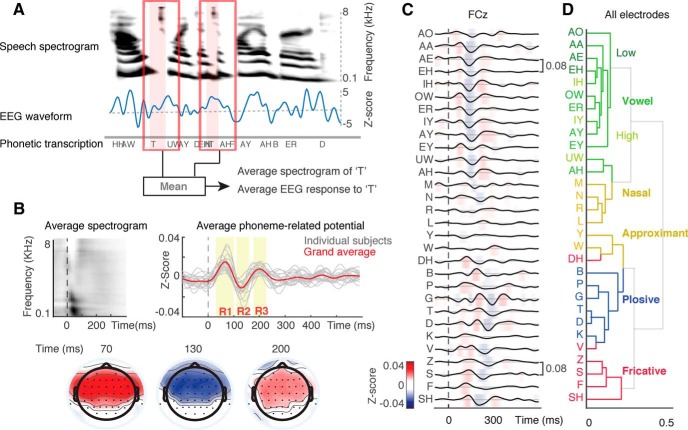
Representation of phonetic information in the PRP. ***A***, PRPs are calculated by averaging the time-aligned neural responses to all instances of a phoneme. ***B***, The average EEG response to all phonemes averaged over all electrodes. Individual subjects are shown in gray; grand average PRP is shown in red. Time points where PRP shows a significant response are shaded in yellow (central electrode Cz, *t* test, multiple-comparison corrected via FDR, *q* < 0.01). The average acoustic spectrogram of all phonemes is shown on the left side. The scalp topographies of three significant time points based on Figure 1*B* including 70, 130, and 200 ms are shown at the bottom. ***C***, Grand average PRPs for 30 American English phonemes. PRPs of a frontocentral electrode FCz are plotted from −100 ms before phoneme onset to 600 ms after phoneme onset. ***D***, Hierarchical clustering of PRPs using all electrodes shows encoding of phonetic information largely driven by manner of articulation, highlighted by different colors.

To obtain a time-locked neural response to each phone, the stimuli were first segmented into time-aligned sequences of phonemes using the Penn Phonetics Lab Forced Aligner Toolkit (Yuan and Liberman, 2008). The EEG data were then segmented and aligned according to phoneme onset (Fig. 1*A*). Response segments where the magnitude exceeded ±10 units were rejected to reduce the effect of biological artifacts, such as eye blinking. On average, 8% of data was removed for each subject. Neural responses within the first 500 ms after the onset of each utterance were not included in the analysis to minimize the effect of onset responses.

PRPs and average auditory spectrograms of phonemes were calculated by averaging the time-aligned data over each phoneme category. Defining *s*(*f*,*t*) as the acoustic spectrogram at frequency *f* and time *t*, and *r*(*e*, *t*) as the EEG response of electrode *e* at time *t*, the average spectrograms and PRP for phoneme *k*, which occurs *N_k_* times and starts at time points of *T_k1_*, *T_k2_*, …,*T_kn_*, are expressed as follows (Eq. 1):

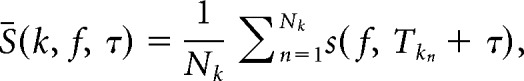


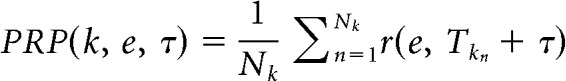
 Where *S̄*(*k*, *f*, τ) is the average auditory spectrogram of phoneme *k*, at frequency *f*, and time τ, and *PRP*(*k*, *e*, τ) is the average response of phoneme category *k*, at electrode *e* and time τ relative to the onset of the phoneme (Mesgarani et al., 2008). As shown in Equation 1, *PRP* is a function of time relative to the onset of phonemes.

To group the PRPs based on their similarity, we performed unsupervised hierarchical clustering based on the unweighted pair group method with arithmetic mean algorithm (Euclidean distance; Jain and Dubes, 1988). To study the separability of different manners of articulation in neural and acoustic space, we used the *F* statistic at each time point to measure the ratio of the distance between and within different manner of articulation groups.

##### Neural representation of acoustic phonetic categories.

Pairwise phoneme distances were estimated using a Euclidean metric (Deza and Deza, 2009) to measure the distance of each phoneme relative to all other phonemes. This analysis results in a two-dimensional symmetric matrix reflecting a pattern of phoneme similarity that can be directly compared with the distance patterns estimated at different time points.

We compared neural versus acoustic organization of phonemes by finding the covariance value between distance matrices in the acoustic and neural signals. The covariance was calculated from only the lower triangular part of the distance matrices to prevent bias caused by the symmetric shape of the matrix. Calculating the covariance values at all time lags in acoustic and neural spaces results in a two-dimensional neural–acoustic similarity measure at all time lags.

In addition to the neural–acoustic covariance matrix, we calculated a neural–neural similarity matrix by comparing the pairwise phoneme distances at different time lags in PRPs.

To visualize the relational organization of PRPs at different time lags, we applied one-dimensional unsupervised multidimensional scaling (MDS) using Kruskal's normalized criterion to minimize stress for one dimension. The MDS was set to zero when no electrode showed a significant response [multiple-comparison corrected via false discovery rate (FDR), *q* < 0.001].

##### Speaker-dependent pairwise phoneme distances.

We calculated the pairwise Euclidean distance of PRPs for each speaker, resulting in a pairwise phoneme distance matrix with four quadrants, where diagonal quadrants represent within-speaker distances and off-diagonal quadrants represent the between-speaker distances. We measured a speaker index by subtracting between-group distances from within-group distances, both in the PRP and spectrogram data. We calculated the correlation between speaker-dependent patterns in neural and acoustic spaces for each time point that yielded a speaker-dependent neural–acoustic correlation matrix (see Fig. 5*A*).

The speaker-dependent encoding (SE) of phoneme category *i* (see Fig. 6*A*) is defined as follows, where the distance matrices can be estimated from either the neural or acoustic representations (Eq. 2):

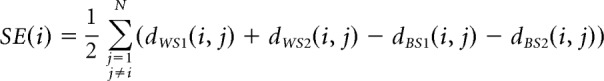
 where *d_ws_*_1_(*i*, *j*) and *d_ws_*_2_(*i*, *j*) are the distances between phonemes *i* and *j* of each speaker (within speaker distances), and *d_BS_*_1_(*i*, *j*) and *d_BS_*_2_(*i*, *j*) are the distances between phoneme *i* and *j* of different speakers (between speaker distances).

## Results

We recorded EEG data from 22 native speakers of American English. Participants listened to simple stories comprising alternating sentences uttered by two speakers (one male, one female). To investigate whether phonemes in continuous speech elicit distinct and detectable responses in the EEG data, we used phonetic transcription of speech data (Yuan and Liberman, 2008) to segment and align the neural responses to all phoneme instances (Fig. 1*A*). We refer to the resulting time-locked evoked responses to phonemes as PRPs. By averaging over all phonemes, we found a robust PRP response at most electrodes. The response of a representative electrode (central electrode Cz) is shown in Figure 1*B*. We applied two-tailed paired *t* test (corrected for FDR; Benjamini and Hochberg, 1995; Benjamini and Yekutieli, 2001; *q* < 0.01) to compare the PRP response with baseline activity. We observed three statistically significant time intervals of 50–90 ms [response (R) 1, positive deflection], 100–160 ms (R2, negative deflection), and 190–210 ms (R3, positive deflection; Fig. 1*B*). The distribution of the PRP across electrodes shows a broadly distributed response strongest in frontocentral electrodes (Fig. 1*B*), a finding consistent with the topographical map of the standard AEP on frontocentral electrodes (Hillyard et al., 1971; Lalor et al., 2009), even though the individual phonemes in continuous speech are not isolated events.

### Encoding of phonetic categories in PRPs

To study whether different phonemic categories elicit distinct neural responses, we averaged the PRP responses over all instances of each phoneme and across all subjects, excluding phoneme categories that contained <0.01% of all phones. Visual inspection of PRPs elicited by each phoneme suggests that they vary in their magnitude and latency, with a varied degree of similarity relative to each other. For example, PRPs for vowels show similar patterns of activation, which differ from that of consonants [Fig. 1*C*, frontocentral electrode (FCz), averaged over all subjects].

To determine whether PRPs can be characterized by phonetic feature hierarchy (Halle and Stevens, 1991; Stevens, 2000), we used an unsupervised clustering method based on the Euclidean distance between PRPs of different phoneme categories. Hierarchical clustering was performed on neural responses over an interval of 0–400 ms after phone onset. This window was chosen to ensure the inclusion of significant components of the average PRP as determined by the statistical analysis shown in Figure 1*B*. The hierarchical clustering reveals different tiers of grouping corresponding to different phonetic features (Fig. 1*D*): the first tier distinguishes obstruent from sonorant phonemes (Ladefoged and Johnson, 2010). Within the obstruent tier, a second tier further differentiates categories based on manner of articulation, where plosives (blue) formed a separate group from the fricative (red) phoneme group. Place of articulation appears in the lower tiers of the hierarchy, separating high vowels from low vowels (Fig. 1*D*, light green for high vowels vs dark green for low vowels). Overall, the clustering analysis of PRPs shows that manner of articulation is the dominant feature expressed in the responses, followed by place of articulation, particularly for vowels. This finding is consistent with neural representation of speech on the lateral surface of the superior temporal gyrus (Chan et al., 2014; Mesgarani et al., 2014), the acoustic correlates of manner and place of articulation features (Stevens, 2000), and psychoacoustic studies showing more confusions among phonemes with the same manner of articulation (Miller and Nicely, 1955; Allen, 1994).

### Time course of phonetic feature encoding in the PRP

**Figure 2.**
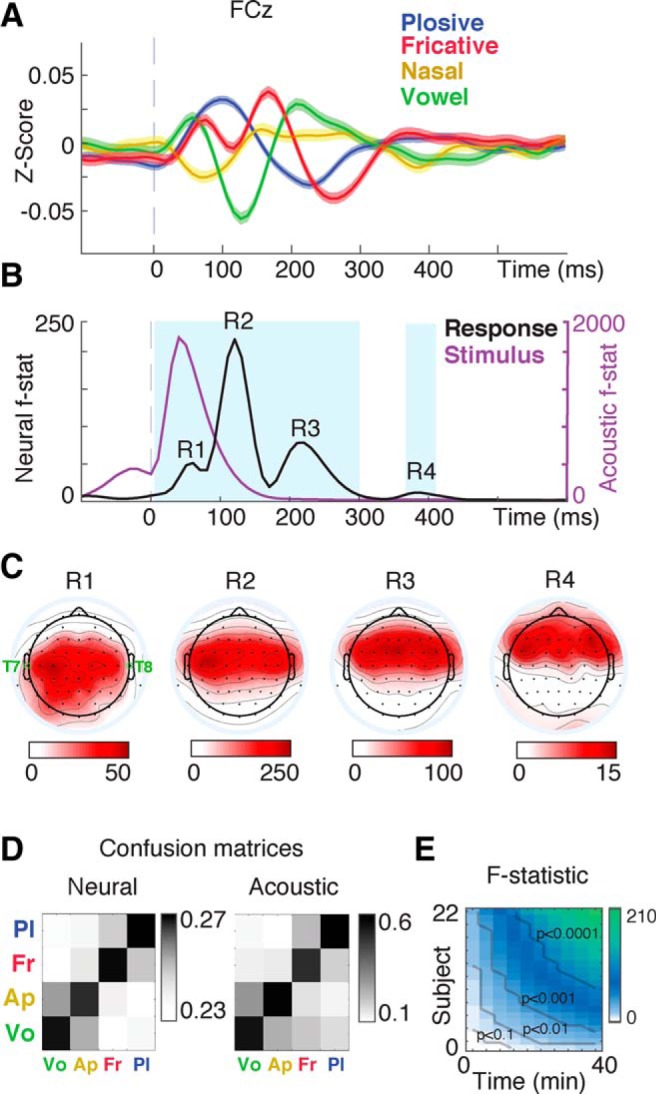
Time course of phonetic feature representation in the PRP. ***A***, The average responses of phonemes that share the same manner of articulation show the time course of manner-specific PRPs (electrode FCz). ***B***, *F* statistic for manner of articulation groups reveals three distinct intervals with significantly separable responses to different manners of articulation (shown in blue, FDR corrected, *q* < 0.05). Neural *F* statistic (black; response) is based on PRP responses recorded from electrode FCz. Acoustic *F* statistic (purple; stimulus) is based on acoustic spectrograms of phonemes. ***C***, Scalp topographies of the *F* statistic calculated by each electrode for the four response events, R1, R2, R3, and R4. The two temporal electrodes of T7 and T8 are marked on the first topographical map. ***D***, Similarity of confusion patterns for manners of articulation for acoustic and neural signals (*r* = 0.59, *p* = 0.016). ***E***, Effect size accumulated over subjects and stimulus duration (*F*-statistic measure for electrode FCz).

To study the temporal characteristics of PRPs, we grouped the PRPs according to the top clusters identified in Figure 1*D*, which also corresponds to the manner of articulation categories of plosives, fricatives, nasals, and vowels (Ladefoged and Johnson, 2010). Each of these phonemic categories have distinctive spectrotemporal properties. For example, plosives have a sudden and spectrally broad onset, fricatives have an energy peak in higher frequencies, and vowels have relatively centered activity at low to medium frequencies. As the vowels become more “front”-ed, the single peak broadens and splits. Compared with vowels, nasals are spectrally suppressed (Ladefoged and Johnson, 2010). The time course of manner-specific PRPs (Fig. 2*A*) shows discrimination between different manners of articulation as early as 10 ms after phoneme onset to as delayed as 400 ms after phoneme onset. As shown in the next section, this early response (R1, 10–50 ms) is mainly due to the structure of the speech stimulus that influences the preceding phonemes. We used the *F* statistic (Patel et al., 1976) to measure the ratio of variance within and between different manners to systematically study the temporal separability of PRPs for different manners of articulation. *F*-statistic analysis reveals significant separability between manners of articulation (Fig. 2*B*; multiple-comparison corrected via FDR, *q* < 0.05) with *F*-statistic peaks observed at four distinct time points (components) centered around 50, 120, 230, and 400 ms. Repeating the same analysis using the acoustic spectrograms (Fig. 2*B*, purple) instead of EEG data (Fig. 2*B*, black) does not show the late response components, validating their neural origin as opposed to possible contextual stimulus effects. Distinct temporal components were also observed in the average PRP with R1 at 70 ms, R2 at 130 ms, and R3 at 200 ms (Fig. 1*B*).

Comparing the *F* statistic and average PRP reveals the unique characteristics of each temporal component. For example, although the first component of PRP (R1) elicits the response with the largest magnitude, it is comparatively less dependent on phoneme category compared with R2 and R3, as evidenced by a smaller *F* statistic. The peak of the *F* statistic indicates that the most phonetically selective PRP response appears at 120 ms (R2). Additionally, the PRP component occurring at 400 ms (R4) in the *F* statistic (Fig. 2*B*) was not apparent in the average PRP (Fig. 1*B*) because the opposite signs of deflection at this time point for different manners (Fig. 2*A*) cancel each other out.

Calculating the *F* statistic between manners of articulation for individual electrodes (Fig. 2*C*) show different scalp maps for early and late PRP components with a varying degree of asymmetry. For example, two temporal electrodes of T7 and T8 show significant discriminability at R2 and R3 but not at R4. It has been shown that cortical sources of ERP responses recorded at T7 and T8, known as the T complex (McCallum and Curry, 1980), are independent from frontocentral activities (Ponton et al., 2002). This suggests that various cortical regions may contribute differently to the response components of R1–R4 in phoneme-related potentials.

To examine both the separation and overlap of different manners of articulation, we trained a regularized least square classifier (Rifkin et al., 2003) to predict the manner of articulation for individual instances of PRPs (10% of data used for cross-validation). The classification accuracy is observed to be significantly above chance for all categories. To compare the confusion patterns (Fig. 2*D*) of manners of articulation in neural and acoustic spaces, we also tested the classification of manners using spectrograms of phones. Figure 2*D* shows that the confusion patterns in neural and acoustic spaces are highly correlated (*r* = 0.59, *p* = 0.016, *t* test), suggesting that the acoustic overlap between various phones is also encoded in the neural responses.

Finally, to determine the variability of PRPs across subjects, we estimated *F* statistics for manners of articulation accumulated over subjects and recording time (Fig. 2*E*). This analysis is particularly informative because it specifies the minimum number of subjects needed to obtain a statistically significant PRP response for a given experimental duration.

### Recurrent appearance of acoustic similarity of phonemes in PRPs

The previous analysis illustrates how phonetic feature categories shape PRPs and their distinct temporal components. However, it does not explicitly examine the relationships between the EEG responses and the acoustic properties of speech sounds. Because speech is a time-varying signal with substantial contextual and duration variability, it is therefore crucial to compare the neural and acoustic patterns over time to control for the temporal variability of phonemes. We therefore used pairwise phoneme similarities calculated at each time point relative to the onset of phonemes, and compared the similarity patterns in neural and acoustic data at each time. As a result, this direct comparison can separate the intrinsic dynamic properties of neural encoding from the temporal dependencies that exist in natural speech. Moreover, this analysis focuses on the encoding of similarities and distinctions between categories rather than the encoding of individual items, and has been widely used in the studies of the visual system to examine representational geometries and to compare models and stages of object processing (Kriegeskorte and Kievit, 2013; Cichy et al., 2014).

**Figure 3.**
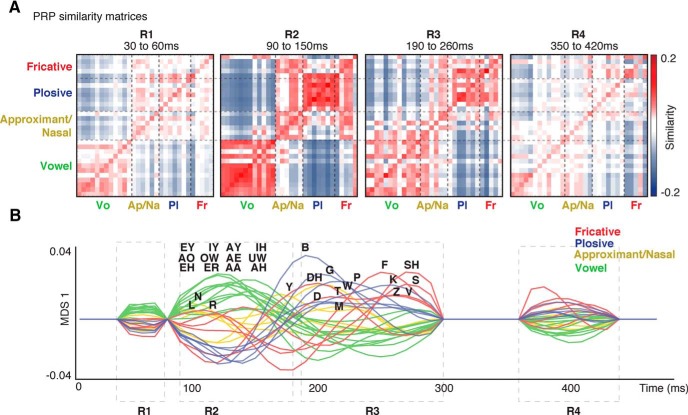
Patterns of phoneme similarity in EEG. ***A***, PRP similarity matrices at time points corresponding to R1, R2, R3, and R4. Similarity is defined as *m* − (*Euclidean distance*) where *m* is the mean value of each matrix. ***B***, One-dimensional representation of the PRP distance matrices at each time point based on electrodes showing significant distinctions between manners of articulation (multiple-comparison corrected via FDR, *q* < 0.001). Time points where no significant electrode was found are set to zero.

We start by calculating the Euclidean distance between the PRPs for each phoneme pair and at every time lag, yielding a time-varying pairwise phoneme distance matrix. We use *m* − *D* as a measure of similarity, where *D* is the distance matrix, and *m* is the mean value of elements of matrix *D*. Figure 3*A* shows the phoneme neural similarity matrices calculated at time points R1, R2, R3, and R4, where red values indicate more similar phoneme pairs (Fig. 3*A*).

To illustrate the temporal progression of relative distances between the PRPs, we used MDS analysis (Borg and Groenen, 2005) and projected the PRP distance matrices at each time lag to a single dimension, where the MDS values are derived from the responsive electrodes at each time point. The MDS result shows the phoneme separability is largest at R2 (Fig. 3*B*; Movie 1). Figure 3*B* also shows the difference in timing of responses to different manners of articulation is most apparent at PRP component R3 compared with R1, R2, and R4 components.

**Figure 4.**
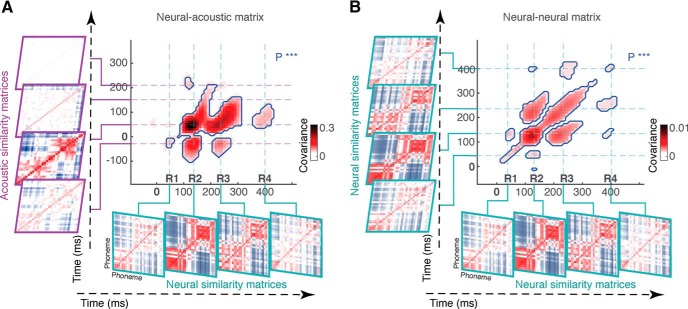
Recurrent appearance of patterns of phoneme similarity in PRPs. ***A***, Neural–acoustic similarity matrix: the covariance between acoustic similarity matrices and PRP similarity matrices at different time lags. The four distinct temporal events are marked as R1, R2, R3, and R4. ***B***, Neural–neural similarity matrix: the covariance of PRP similarity matrices at different time lags.

To compare phoneme similarity patterns in acoustic and neural data over time, we calculated the acoustic similarity matrix using the acoustic spectrogram of phones (Yang et al., 1992) and found the covariance between the corresponding similarity matrices. The covariance values (Fig. 4*A*, neural–acoustic matrix) demonstrate distinct time intervals when the organization of phonemes in PRP mirrors the acoustic organization of phonemes (significance was assessed using bootstrapping, *n* = 20, multiple-comparison corrected via FDR, *q* < 0.0001). In particular, the acoustic distance matrix at time interval 10–60 ms is significantly similar to the neural data at three time intervals, approximately centered at 120 (R2), 230 (R3), and 400 ms (R4) after the phoneme onset. R1 (40 ms) in neural data, on the other hand, is similar to acoustic patterns at ∼−30 ms, showing that the observed distinctions between phonemes at R1 are mainly caused by the acoustic structure of the preceding phonemes. We also calculated the covariance between PRP distance matrices at different time lags (Fig. 4*B*; neural–neural matrix, bootstrapping, *n* = 20, multiple-comparison corrected via FDR, *q* < 0.0001). Figure 4*B* shows that the PRP similarity matrix at R3 is significantly similar to the similarity matrices at R2 and R4. The main diagonal of neural–neural covariance matrix demonstrates the start and ending of the significant PRP responses, as well as the strength of phoneme distinction at each duration. In summary, Figure 4 shows that the organization of neural responses at time intervals R2, R3, and R4 mirrors the acoustic similarities of different phonemes, and provides compelling evidence for a repetitive appearance of acoustic phonetic distinctions in the neural data.

### Encoding of speaker characteristics in PRPs

**Figure 5.**
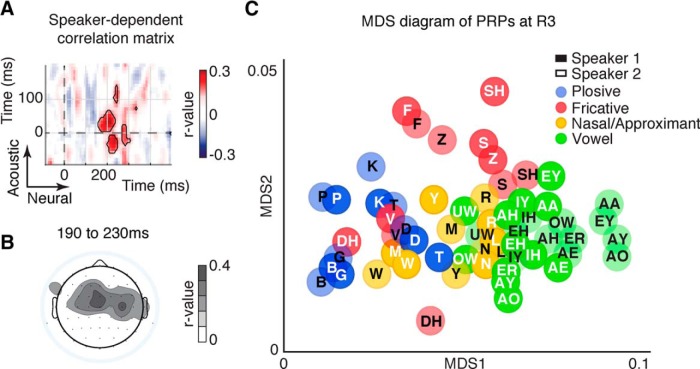
Encoding of speaker characteristics in PRPs. ***A***, Correlation between speaker-dependent pairwise phoneme distances in neural space and acoustic space. Maximum correlation occurs ∼200 ms after phone onset, corresponding to R3. The black contour indicates significant correlation (FDR corrected, *q* < 0.05). ***B***, Scalp topography showing average speaker-dependent correlation from 190 to 230 ms. ***C***, Two-dimensional MDS diagram of PRPs. Colored bubbles show manner of articulation; black letters denote speaker 1; white letters denote speaker 2.

The previous analysis showed that the encoding of phonetic distinctions in the PRPs can be directly related to the acoustic characteristics of phonetic categories. However, in addition to deciphering the semantic message encoded in the speech signal, a listener also attends to acoustic cues that specify speaker identity. To study whether the variations in acoustic cues of different speakers is encoded in the PRP, we modified the pairwise phoneme similarity analysis (Fig. 5*A*) by estimating the pairwise distances between phonemes of each speaker and between phonemes of different speakers. To measure speaker dependency of EEG responses, we subtracted the sum of the pairwise phoneme distances for each speaker and across speakers, an approach that highlights the PRP components that show a differential response between the two speakers. The correlations between the speaker distance matrices in acoustics and the speaker distance matrices in PRPs are shown in Figure 5*A*, where the most significant resemblance between speaker-dependent matrices occurs at R3 (200 ms, *r* = 0.46, *p* < 0.01). This observation differs from the timing of the largest phonetic distinctions in the PRP observed at R2 (compare Figs. 4*A*, 5*A*), showing significant time differences in the encoding of different acoustic features. The scalp location of speaker feature encoding is shown in Figure 5*B*.

We used a multidimensional scaling analysis to visualize the relative distance of the PRPs estimated separately for each speaker. As shown in Figure 5*C*, speaker-dependent characteristics (indicated by white and black fonts) are secondary to phonetic features (indicated by colored bubbles), meaning that the phonetic feature distinctions in the PRP are greater than speaker-dependent differences. We quantified this preferential encoding using a silhouette index (Rousseeuw, 1987), and found a silhouette index significantly greater for the PRP clusters corresponding to manner of articulation compared with the PRP clusters that represent speaker differences (silhouette index, 0.18 vs 0.001).

**Figure 6.**
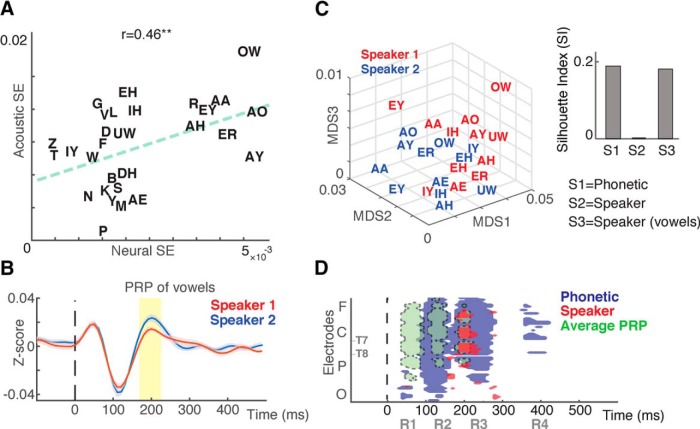
Joint neural encoding of speaker and phonetic features. ***A***, Significant correlation between speaker feature information encoded in PRPs and acoustic spectrograms. ***B***, Average PRPs corresponding to vowels articulated by speaker 1 versus vowels articulated by speaker 2. SE is shown by shaded color based on different subjects. ***C***, Three-dimensional MDS diagram showing the separation of PRPs between the two speakers. Silhouette index quantifies the clustering of PRPs using the following: S1, separation of manners of articulation in all PRPs; S2, separation of speakers in all PRPs; and S3, separation of speakers within group of vowels. ***D***, Comparison of timing and the location of response for average PRP, response components correlated with acoustic discriminability of phoneme categories, and response components correlated with speaker differences. Electrodes proceed in the anterior direction in rows from left to right and are ordered as follows: frontal (F), central (C), parietal (P), occipital (O). Temporal electrodes are marked with T7 and T8.

Next, we wanted to examine the encoding of the variable degree of acoustic similarity between different phonemes of the two speakers. This varied acoustic similarity is caused by the interactions between the physical properties of the speakers' vocal tracts and the articulatory gestures made for each phoneme. To test the dependence of speaker representation in neural responses on different phonemes, we defined an index (SE) that measures the response similarity between the phonemes of the two speakers. Therefore, this index would be zero if the responses to the same phonemes of two speakers were identical. We compared speaker-dependent phoneme distances in acoustic and neural signals (Fig. 6*A*, *r* = 0.46, *p* < 0.014), where the high correlation value implies a joint encoding of speaker–phoneme pairs. Our analysis shows that the separation between the two speakers is higher in the group of vowels. To more explicitly study speaker representation of vowels, we found the average PRPs for vowels for each of the two speakers. The average vowel PRPs of the two speakers show a significant separation at ∼200 ms after the phoneme onset (corresponding to R3; Fig. 6*B*). To visualize vowel separation at this time interval, we used a three-dimensional MDS diagram (Fig. 6*C*), where the separation between the two speakers is readily observable. We quantified the separation of speakers within the group of vowels using the silhouette index (Rousseeuw, 1987; Fig. 6*C*, S3), which revealed greater separation within the group of vowels compared with the separation of speakers in all PRPs.

Finally, Figure 6*D* summarizes the scalp location and timing for the three main analyses in our study: (1) the average PRP of all phonemes (Fig. 1*B*), (2) response components corresponding to acoustic phoneme similarity patterns (Fig. 4*A*), and (3) response components correlated with speaker differences (Fig. 5*A*). The largest average PRP component appears at R1, maximum phonetic distinctions are encoded at R2, and speaker dependency was best represented at R3.

## Discussion

We observed that EEG responses to continuous speech reliably encode phonetic and speaker distinctions at multiple time intervals relative to the onset of the phonemes. The responses are primarily organized by phonetic feature, while subtler speaker variations appear within manner of articulation groups, consistent with previous studies showing a larger role for phonetic over speaker characteristics in shaping the acoustic properties of phones (Syrdal and Gopal, 1986; Johnson, 2008).

Our finding of repetitive appearance of phonetic distinction in the neural response is consistent with AEP studies of isolated consonant–vowel pairs (Picton et al., 1977; Näätänen and Picton, 1987; Ceponiene et al., 2002; Tremblay et al., 2003; Martin et al., 2008). However, relating the PRP components (R1–R4) to specific AEP events, such as the P1–N1–P2 or N2–P3–N4 complex, requires further investigation. Making this comparison is challenging because of the differences in the shape of PRP and AEP responses, including the sign of the deflection. For example, the sign of PRP deflection for different manner groups is not always positive–negative–positive, as is the case in AEP. In particular, R2 deflection is positive for plosive phonemes and negative for the vowels. Possible reasons for the observed differences between AEP and PRP is the dominance of onset response in AEP, in addition to contextual effects that may influence the average responses to a particular phoneme. In addition, continuous speech is likely to engage higher-level, speech-specific regions that may not be activated when a person hears isolated consonant–vowel tokens (Honey et al., 2012; Overath et al., 2015).

While our observation of scalp distributions at each time point suggests a different underlying pattern of neural activity for each component, the neural sources contributing to R1–R4 remain unclear. Studies have shown that AEPs can be subdivided into three categories: (1) responses with latency <10 ms are associated with brainstem; (2) response latencies between 10 and 50 ms are associated with thalamic regions; and (3) response latencies beyond 50 ms are mostly generated by cortical regions (Liégeois-Chauvel et al., 1994; Picton et al., 2000). Within cortical responses, comparison of high-gamma and AEP (Steinschneider et al., 2011a, 2011b), as well as attention and development studies (Picton and Hillyard, 1974; Pang and Taylor, 2000; Crowley and Colrain, 2004; Kutas and Federmeier, 2011), has shown that different cortical regions are responsible for generating P1, N1, P2, N2, and N4. Based on these findings, it is possible that the diverse timing of the observed components of PRP could be the combined effect of the activity of several cortical regions. The pairing of source connectivity analysis along with complementary neuroimaging techniques should allow for more detailed characterizations of neural processes in future studies (Schoffelen and Gross, 2009). Additionally, the systematic manipulation of the stimulus, task, and behavior may yield better characterization of the sensory and perceptual processes contributing to the representation of the acoustic features we observed at different time intervals (Friederici et al., 1993; Kaan et al., 2000; Patel, 2003).

One major difference between our study and previous work is the direct comparison between the organization of neural responses and acoustic properties of speech sounds. Therefore, the neural encoding of acoustic features can be investigated at each time point that may represent the underlying stages of cortical processing. In contrast with regression-based approaches (Di Liberto et al., 2015), which average neural responses over the duration of phonemes, our approach maintains the precise temporal features of the neural response. Our results lay the groundwork for several research directions, for example, where explicit changes in the representational properties of speech can be examined in speech development (Dehaene-Lambertz et al., 2002), in phonotactic probabilities in speech (Vitevitch and Luce, 1999), in contexts where a listener learns new acoustic distinctions (Logan et al., 1991; Polka and Werker, 1994), in second-language acquisition (Ojima et al., 2005; Rossi et al., 2006), and in changes in the representational properties of speech through varying task demands (Mesgarani and Chang, 2012). Given that the N1 and P1 sequences in AEP are not fully matured in children and teenagers, it remains to be seen how this can change the PRP components we report in this paper (Pang and Taylor, 2000; Ceponiene et al., 2002; Wunderlich and Cone-Wesson, 2006). The ability to directly examine the representational properties of the spoken language stimulus in neural responses is a powerful tool for distinguishing among the many factors involved in sensory processing (Luck, 1995; Thierry et al., 2007). For example, speech and communication disorders can stem from a loss of linguistic knowledge or from a degraded representation of relevant acoustic cues, such as in disorders of the peripheral and central auditory pathways. The source of the problem is unclear for speech disorders, such as aphasia (Kolk, 1998; Swaab et al., 1998; ter Keurs et al., 2002). Since phoneme-related potentials can track the representational properties of speech as it is processed throughout the auditory pathway, these potentials could be instrumental in comparing healthy and disordered brains and identifying possible problem sources.
